# Microstructural Variations in Laser Powder Bed Fused Al–15%Fe Alloy at Intermediate Temperatures

**DOI:** 10.3390/ma15134497

**Published:** 2022-06-26

**Authors:** Wenyuan Wang, Naoki Takata, Asuka Suzuki, Makoto Kobashi, Masaki Kato

**Affiliations:** 1 Department of Materials Process Engineering, Graduate School of Engineering, Nagoya University, Furo-cho, Chikusa-ku, Nagoya 464-8603, Japan; suzuki.asuka@material.nagoya-u.ac.jp (A.S.); kobashi.makoto@material.nagoya-u.ac.jp (M.K.); 2 Aichi Center for Industry and Science Technology, 1267-1 Akiai, Yakusa-cho, Toyota 470-0356, Japan; masaki_2_katou@pref.aichi.lg.jp

**Keywords:** additive manufacturing, aluminum alloys, microstructure, intermetallics, thermal exposure

## Abstract

The samples of the Al–15Fe (mass%) binary alloy that were additively manufactured by laser powder bed fusion (L-PBF) were exposed to intermediate temperatures (300 and 500 °C), and the thermally induced variations in their microstructural characteristics were investigated. The L-PBF-manufactured sample was found to have a microstructure comprising a stable θ-Al_13_Fe_4_ phase localized around melt-pool boundaries and several spherical metastable Al_6_Fe-phase particles surrounded by a nanoscale α-Al/Al_6_Fe cellular structure in the melt pools. The morphology of the θ phase remained almost unchanged even after 1000 h of exposure at 300 °C. Moreover, the nanoscale α-Al/Al_6_Fe cellular structure dissolved in the α-Al matrix; this was followed by the growth (and nucleation) of the spherical Al_6_Fe-phase particles and the precipitation of the θ phase. Numerous equiaxed grains were formed in the α-Al matrix during the thermal exposure, which led to the formation of a relatively homogenous microstructure. The variations in these microstructural characteristics were more pronounced at the higher investigated temperature of 500 °C.

## 1. Introduction

Metal additive manufacturing (AM) is an advanced manufacturing technology used for fabricating complex-shaped metal/alloy components using computer-aided design [1]. Laser powder bed fusion (L-PBF) is one of the representative metal-AM processes [2] that has been adopted for a considerably lightweight aluminum (Al) alloy series [3,4]. In L-PBF, a scanning laser irradiates the metal/alloy powder that is sequentially bedded on a base plate, which leads to selective melting and subsequent rapid solidification. Consequently, L-PBF-processed Al alloy products exhibit significantly refined microstructures [5,6,7,8], which lead to higher strengths than those of the Al alloys fabricated using conventional casting processes [9,10,11,12]. Al–Si-based alloys are known to be suitable for the L-PBF process [13,14,15]; however, L-PBF-processed Al–Si-based alloys exhibit reduced strength at temperatures higher than 200 °C [16]. The potential application of L-PBF-manufactured Al alloys in radial impellers operating at intermediate temperatures above 200 °C (inside the vehicle turbochargers) has encouraged the development of new Al alloys with superior strength at both ambient and intermediate temperatures. To accommodate the demand for materials with high-temperature strength, a variety of heat-resistant Al alloys, such as Al–Cr, Al–Mn, Al–Ni, Al–Ni–Fe, and Al–Ce–Mn, have been proposed for fabrication by L-PBF [17,18,19].

With the aim of fabricating Al alloys using common alloy elements instead of rare-earth elements, attempts have been made to investigate the feasibility of adopting L-PBF to fabricate an Al–Fe binary alloy with a high Fe content (15 mass%) [19], which corresponds to a hyper-eutectic composition in the Al–Fe binary system. In general, coarsened Al–Fe intermetallic compounds (stable Al_13_Fe_4_ phase) were often formed in the cast Al–Fe-based alloys with high Fe contents. The brittle Al-rich intermetallic phases have a detrimental effect on the ductility of the materials. However, the L-PBF-manufactured Al–15%Fe alloy exhibits refined microstructures [20,21] containing numerous nanosized particles of the metastable Al_6_Fe phase [22]. Moreover, the L-PBF-manufactured Al–15%Fe alloy shows a high yield strength of about 400 MPa at 300 °C [23], which is higher than that of both the 8xxx alloy series [24,25] (Al–Fe-based alloys used in powder metallurgy) and the L-PBF-manufactured Al-based multi-element alloys [18,26]. The hardness of these specimens slightly decreases after long-term thermal exposure, suggesting that the high thermal stability of the nanosized metastable Al_6_Fe phase strengthens the L-PBF-manufactured Al–Fe alloys. This indicates the remarkable potential of Al–15%Fe as a lightweight Al alloy that can be additively manufactured for high-temperature applications. However, the metastable Al_6_Fe phase can transform into a stable Al_13_Fe_4_ phase [27] that is in equilibrium with the α-Al matrix after long-term exposure to high temperatures. Additionally, the changes in the microstructural features of L-PBF-processed Al–Fe binary alloys during thermal exposure are not fully understood.

Therefore, the microstructures of the L-PBF-processed Al–15%Fe alloy exposed to intermediate temperatures (300 and 500 °C), which contained refined Al–Fe intermetallic phases, were systematically characterized in this study to elucidate the microstructural variations and the phase transformation from metastable Al_6_Fe to stable Al_13_Fe_4_.

## 2. Materials and Methods

An Al–15Fe (mass%) binary alloy powder with an average particle size below 30 μm was prepared via gas atomization; the details concerning the preparation of the alloy powder can be found elsewhere [20]. Rectangular samples with the approximate dimensions of 15 × 15 × ~5 mm^3^ were constructed using a ProX DMP 200 machine (3D Systems, Rock Hill, SC, USA). The following optimized L-PBF parameters were used to manufacture the samples [14]: laser scanning speed, 0.4 m/s; laser power, 128 W; hatch distance, 0.1 mm; powder bed layer thickness, 0.03 mm; and beam focus size, ~0.1 mm. The scanning laser patterns were separated in each 10 mm-sized hexagonal grid, and the direction of the laser scanning was consecutively altered by 90° for each powder layer [28]. The constructed samples had high relative densities (>96%). The as-built samples were exposed to 300 and 500 °C for various periods ranging from 1 h to 1000 h, followed by a water quench.

The samples exposed to high temperatures for different durations were embedded in resin and then mechanically polished with SiC paper. Scanning electron microscopy (SEM; JSM-IT500 and JSM-6610A, JEOL Ltd., Tokyo, Japan) was performed to examine the microstructures of the prepared samples. To facilitate these observations, the sample surfaces were polished with 0.05 µm-sized colloidal silica particles (pH 9.8 in liquid). Vickers hardness tests were performed on these samples using a test load of 1.98 N and a loading duration of 15 s at room temperature. X-ray diffraction (XRD) measurements were carried out using a Rigaku ULTIMA IV instrument equipped with a Cu radiation source at 40 kV. The cross-sectional samples were subjected to argon-ion polishing using a cross-section polisher at 6 V. Orientation analyses were performed using the electron backscatter diffraction (EBSD) technique with scanning step sizes of 0.2 or 0.3 μm. Thin samples were prepared from the thermally exposed specimens for transmission electron microscopy (TEM) analysis. The pieces were cut into a plate shape using a low-speed cutter and then polished with SiC paper to prepare foil samples with a thickness of ~0.1 mm. The thin-foil samples were subjected to argon-ion polishing at 6.0 kV using an Ion Slicer™ (JEOL EM-09100IS) and then smoothened for approximately 600 s at a low voltage (2.0 kV) for TEM analysis. The microstructures of the prepared samples were characterized by TEM (JEOL JEM-2100F/HK) at 200 kV.

## 3. Results and Discussion

### 3.1. Microstructure of As-Built Sample

**Figure 1:**
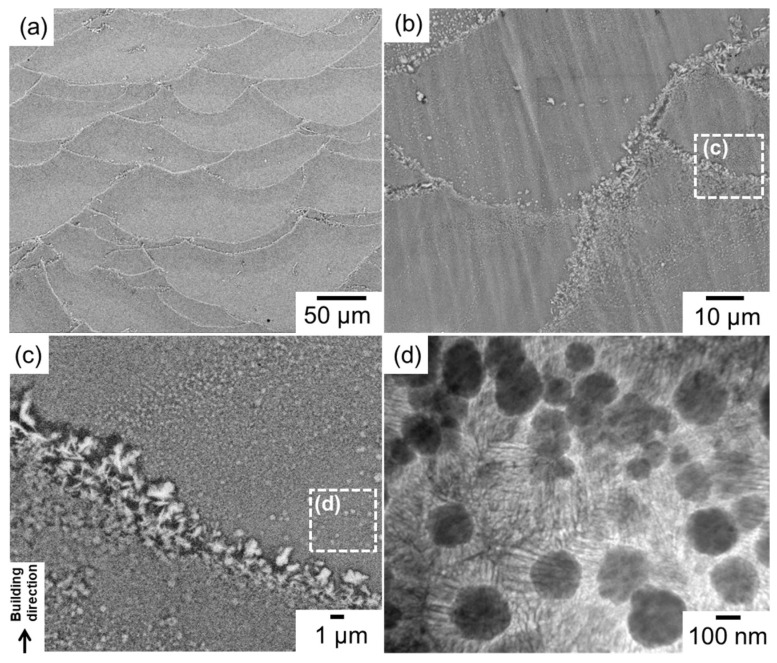
(**a**–**c**) SEM images showing microstructures of L-PBF-constructed Al–15%Fe alloy: (**a**,**b**) low-magnification views and (**c**) a location around the melt-pool boundary. (**d**) Bright-field TEM image showing spherical Al_6_Fe-phase particles surrounded by nanoscale cellular structures in the melt pool.

Figure 1 shows the multi-scale microstructural characteristics of the as-built Al–15%Fe alloy sample. The low-magnification SEM images [Figure 1a,b] show representative melt-pool structures, which refer to the laser-scanning tracks in which the regions are selectively melted and rapidly solidified. The high-magnification SEM image (Figure 1c) shows many coarse particles of the Al–Fe intermetallic phase, with a leaf-shaped morphology localized along the melt-pool boundaries. Comprehensive EBSD analyses [21] have confirmed the formation of the θ-Al_13_Fe_4_ stable phase [27] that is in equilibrium with the α-Al matrix in the Al–Fe binary system. Numerous particles of the metastable Al_6_Fe phase [22], several hundred nanometers in size, were distributed in the melt pools (Figure 1c,d). The TEM observation (Figure 1d) revealed that these spherical Al_6_Fe-phase particles were surrounded by nanoscale cellular structures composed of α-Al and Al_6_Fe phases in the melt-pool structure. The formation sequences of the nanosized metastable phase and the relatively coarse stable phase during solidification have been clarified [21] using the equilibrium and non-equilibrium phase diagrams of the Al–Fe system [29].

### 3.2. Variation in Al–Fe Intermetallic Phases at Elevated Temperatures

**Figure 2:**
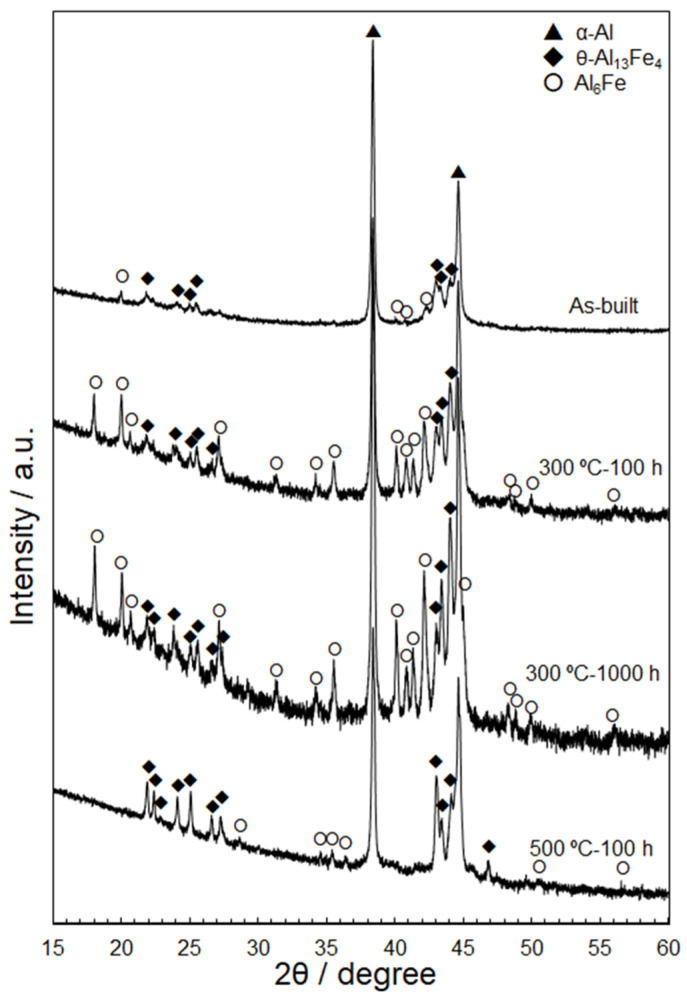
X-ray diffraction (XRD) profiles of the L-PBF-built pristine and thermally exposed Al–15%Fe alloy samples [300 °C (100 h, 1000 h) and 500 °C (100 h)].

Figure 2 shows representative XRD profiles of the as-built and thermally exposed Al–15%Fe alloy samples. The XRD profile of the as-built sample confirmed the presence of metastable Al_6_Fe and stable θ-Al_13_Fe_4_ phases in the α-Al matrix, which is consistent with the microstructural characterization results shown in Figure 1. The samples exposed to 300 °C exhibited higher diffraction intensities from the Al_6_Fe and θ phases; this tendency was enhanced after the long-term exposure for 1000 h. In contrast, the sample exposed to 500 °C for 100 h exhibited considerably lower diffraction intensities from the Al_6_Fe phase and similar θ-phase intensities to those detected in the samples exposed to 300 °C. This variation in the XRD profiles can be attributed to the dissolution of the metastable Al_6_Fe phase upon exposure to 500 °C.

**Figure 3:**
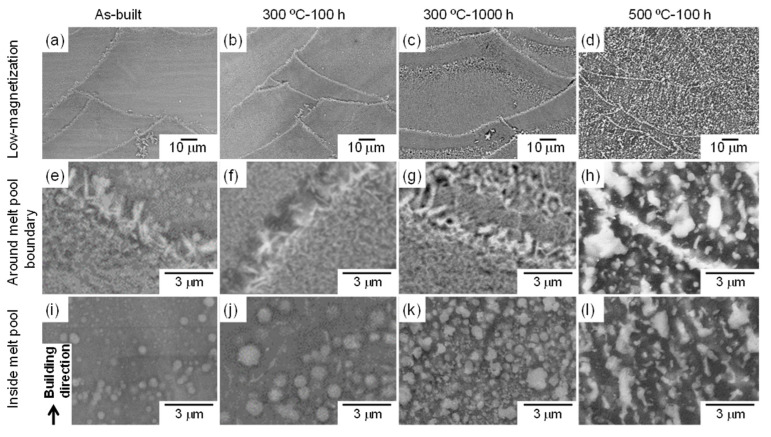
SEM images showing microstructures of (**a**,**e**,**i**) pristine and (**b**–**d**,**f**–**h**,**j**–**l**) thermally exposed L-PBF-built Al–15%Fe alloy samples: (**b**,**f**,**j**) 300 °C/100 h, (**c**,**g**,**k**) 300 °C/1000 h, and (**d**,**h**,**l**) 500 °C/100 h.

Figure 3 shows the SEM-EBSD images that reveal the microstructures at different locations in the as-built and thermally treated samples (300 and 500 °C). The macroscopic melt-pool structure in the L-PBF-built sample changed minimally upon exposure to 300 °C, even after 1000 h [Figure 3a,c]. Moreover, the stable θ-Al_13_Fe_4_ phase that was localized along the melt-pool boundaries remained unchanged after 1000 h of exposure [Figure 3e,g]. Additionally, the size of the metastable Al_6_Fe-phase particles located in the melt pools increased after 100 h of exposure [Figure 3i,j]; moreover, the number density increased considerably after 1000 h (Figure 3k). The larger volume of the Al_6_Fe phase is consistent with the high diffraction intensities observed in the XRD profiles (Figure 2). These results demonstrate the nucleation and growth of the spherical Al_6_Fe-phase particles after exposure to 300 °C. The quantitative analyses for the spherical Al_6_Fe phase were described elsewhere [23]. However, the spherical Al_6_Fe phase was scarcely found in the sample exposed to 500 °C for 100 h. Although macroscopic melt-pool structures were observed (Figure 3d), abundant coarse intermetallic-phase particles were present in the melt pools (Figure 3l) and at the melt-pool boundaries (Figure 3h). The initial leaf-shaped morphology of the θ phase transformed into a granular or plate-shaped morphology. These granular Al–Fe intermetallic phases were found in the melt pools.

**Figure 4:**
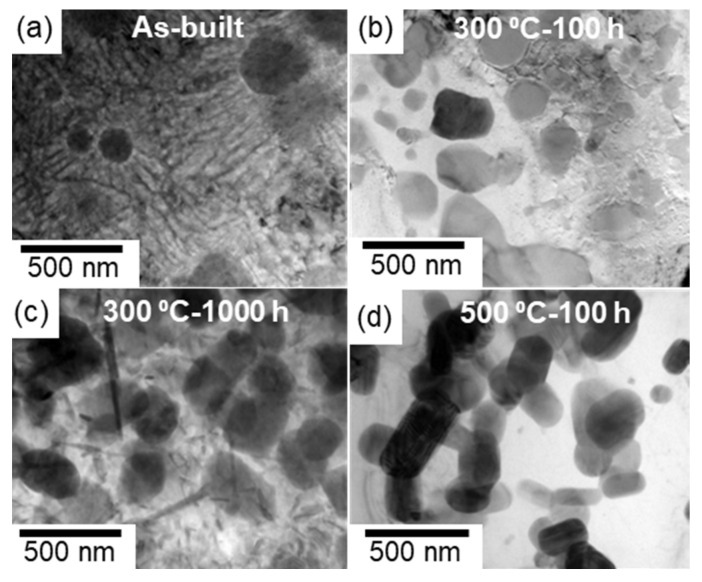
TEM images showing microstructures in the melt pools of the (**a**) as-built and (**b**–**d**) thermally treated L-PBF-built Al–15%Fe alloy samples: (**b**) 300 °C/100 h, (**c**) 300 °C/1000 h, and (**d**) 500 °C/100 h.

Figure 4 shows the bright-field TEM images that reveal the microstructures in the melt pools of the as-built and thermally treated samples (300 and 500 °C). The nanoscale cellular structure of the eutectic α-Al/Al_6_Fe phases, which appeared in the as-built sample, was scarcely observed in the sample exposed to 300 °C for 100 h [Figure 4a,b]; however, various spherical Al_6_Fe-phase particles, several hundred nanometers in size, were observed. After 1000 h of exposure, numerous plate-shaped precipitates and spherical Al_6_Fe-phase particles were found in the α-Al matrix (Figure 4c). These fine precipitates are consistent with the SEM observations of the fine particles (Figure 3k). However, the plate-shaped precipitates were not observed after the exposure to 500 °C (Figure 4d). Moreover, many granular particles appeared to be connected to each other in the α-Al matrix.

**Figure 5:**
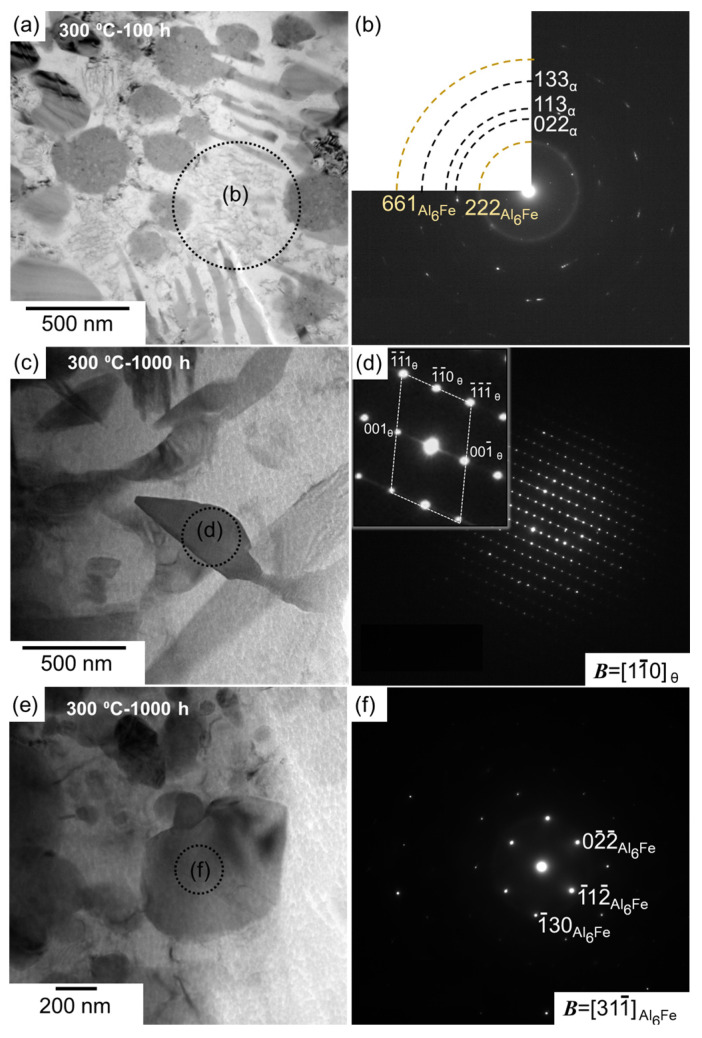
(**a**,**c**,**e**) Bright-field TEM images and (**b**,**d**,**f**) the corresponding SAED patterns of (**a**,**b**) pristine and (**c**–**f**) thermally exposed L-PBF-built samples (300 °C/1000 h).

**Figure 6:**
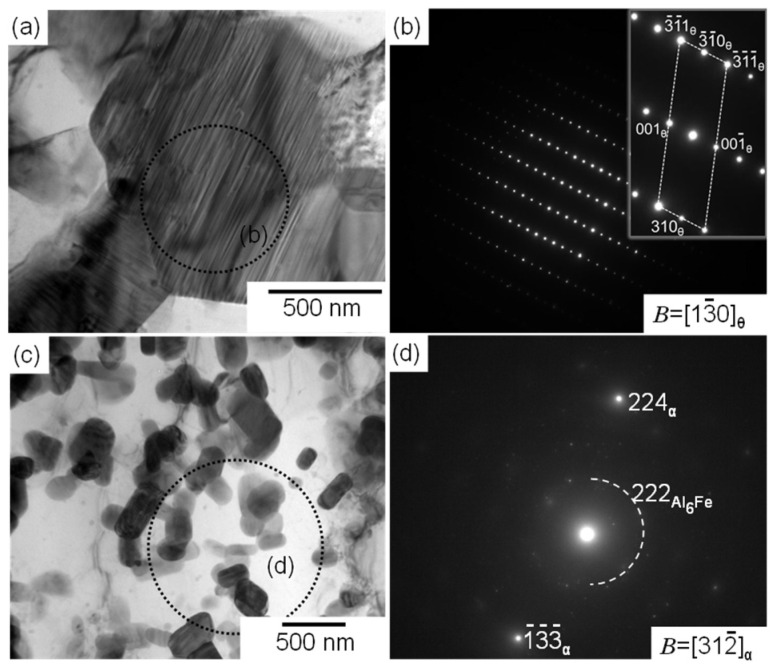
(**a**,**c**) Bright-field TEM images showing the intermetallic phases distributed in the melt pool and the corresponding (**b**,**d**) SAED patterns of an L-PBF-built Al–15%Fe alloy sample exposed to 500 °C for 100 h.

Selected area electron diffraction (SAED) patterns were subsequently acquired to identify the phases observed in the thermally exposed samples; the representative results are summarized in Figure 5 and Figure 6. Each SAED pattern was captured from enclosed areas in the corresponding TEM images. For the coarse cellular structures that were locally observed in the sample exposed to 300 °C for 100 h (Figure 5a), the SAED pattern exhibited a ring diffraction configuration derived from the (222) plane of the Al_6_Fe phase, indicating the presence of a relatively coarsened eutectic structure of the α-Al and Al_6_Fe phases (Figure 5b). The SAED pattern of the sample exposed to 300 °C for 1000 h (Figure 5c), which was acquired from a relatively coarse precipitate with a plate-shaped morphology, as shown in Figure 4c, indicated that the incident beam was parallel to the [1,2,3,4,5,6,7,8,9,10] direction of the θ-Al_13_Fe_4_ stable phase with a monoclinic structure (Figure 5d). The observed morphology of the θ phase is consistent with that of the Al–2.5%Fe alloy manufactured by L-PBF [30]. The SAED pattern captured from the spherical particles that were several hundred nanometers in size (Figure 5e) revealed a clear diffraction pattern derived from the Al_6_Fe phase (Figure 5f). The presence of the spherical Al_6_Fe metastable phase is consistent with the XRD results (Figure 2). Numerous granular intermetallic phases, including several planar faults, were observed in the sample exposed to 500 °C for 100 h (Figure 6a). The corresponding SAED pattern displayed several diffractions derived from the θ phase, and the observed planar faults appeared to be parallel to the (001) plane of the θ phase (Figure 6b). These crystallographic features correspond well to the nanosized twins on the (001) plane in the grown θ phase [31,32]. These results clearly indicate the formation of a coarse stable θ phase in equilibrium with the α-Al phase. Additionally, the granular Al_6_Fe phases remained localized, even after exposure to 500 °C for 100 h (Figure 6c,d).

The aforementioned results indicate that in the samples exposed to 300 °C, the fine metastable Al_6_Fe-phase particles in the eutectic cellular structure dissolve in the α-Al matrix; this is followed by the growth of the spherical metastable Al_6_Fe-phase particles and the precipitation of the stable θ phase in the α-Al matrix. The growth of the stable θ phase is more pronounced at 500 °C. Moreover, the interface between the θ and Al_6_Fe phases was scarcely observed in the samples exposed to the different temperatures, suggesting that the stable θ phase nucleated in the α-Al matrix containing the Fe solute, rather than at the interface of the metastable Al_6_Fe phase with the α-Al matrix.

**Figure 7:**
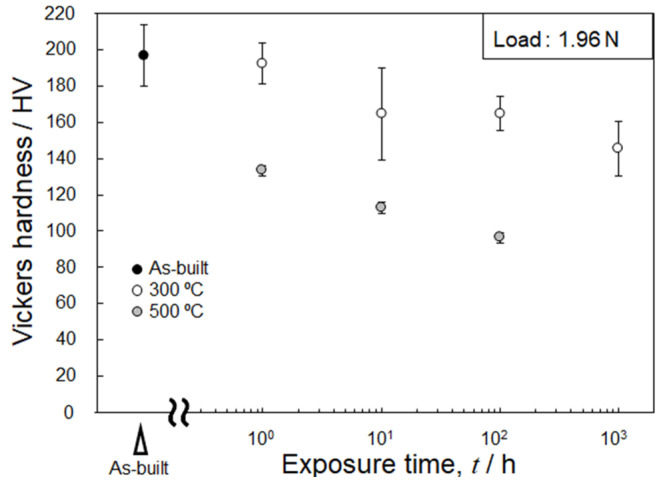
Changes in hardness of the L-PBF-built Al–15%Fe alloy samples as a function of exposure time at 300 and 500 °C.

Figure 7 shows the changes in the Vickers hardness of the L-PBF-built Al–15%Fe alloy samples after the exposure to different temperatures. The as-built sample exhibited a high hardness of ~200 HV. The hardness decreased moderately with the increasing duration of the exposure to 300 °C. The slight reduction in hardness of these specimens is consistent with the gradual microstructural changes observed by SEM and TEM (Figure 3 and Figure 4). However, the hardness of the specimen exposed to 500 °C significantly decreased to below 140 HV after 1 h and then continuously decreased to ~100 HV. This tendency is in good agreement with the significant change in microstructural features observed in Figure 3 and Figure 4 through the replacement of the metastable Al_6_Fe phase with the stable θ phase.

### 3.3. Changes in α-Al Matrix at Elevated Temperatures

**Figure 8:**
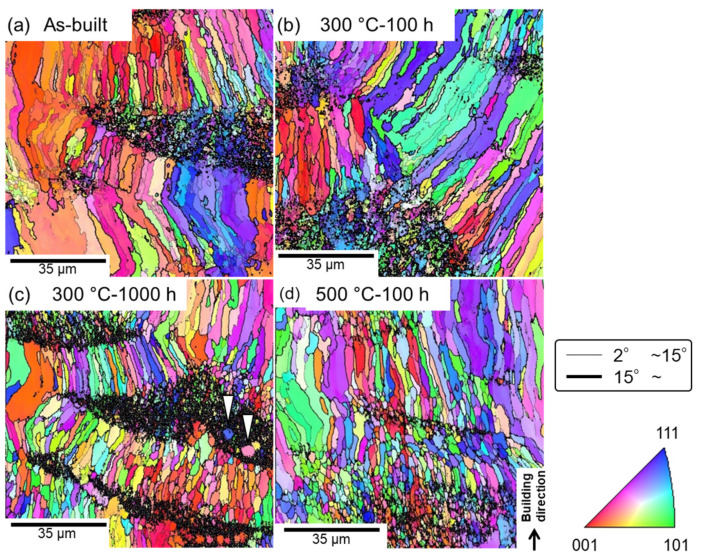
EBSD orientation maps of the fcc-structured α-Al matrix in the (**a**) as-built and (**b**–**d**) thermally exposed samples: (**b**) 300 °C/100 h, (**c**) 300 °C/1000 h, and (**d**) 500 °C/100 h. The fine lines correspond to misorientations (θ) of 2° < θ < 15°, whereas the bold lines represent θ > 15°.

To examine the variations in the microstructural characteristics of the α-Al matrix in the specimens during their high-temperature exposure, the as-built and thermally exposed samples were subjected to an EBSD analysis. Figure 8 shows the orientation distribution maps of the fcc-structured α-Al matrix in the as-built and thermally treated specimens. In these maps, orientations parallel to the build direction are colored according to the attached unit triangle of the inverse pole figure. Several elongated grains with widths of a few tens of micrometers were predominantly formed in the as-built sample and were surrounded by high-angle grain boundaries with large misorientations (>15°); fine-grained microstructures (including unanalyzed areas) were also locally observed (Figure 8a). A comparison of the SEM-observed locations with the EBSD-analyzed areas revealed that the fine-grained regions were typically located inside the melt pools. The finely solidified Al_6_Fe phases likely promoted nucleation during the solidification, resulting in local formation of the fine-grained α-Al phase. These microstructural morphologies remained almost unchanged even after 1000 h of exposure at 300 °C [Figure 8b,c]; nevertheless, relatively equiaxed grains were often observed. Additionally, the density of the low-angle boundaries (with misorientations smaller than 15°) decreased after the exposure. It is worth noting that a few equiaxed grains, several micrometers in size, were formed inside the local fine-grained regions (indicated by the arrowheads in Figure 8c), suggesting the occurrence of recrystallization at elevated temperatures. This trend was more evident in the sample exposed to 500 °C (Figure 8d), which resulted in the formation of a relatively homogenous microstructure in the α-Al matrix. The grain size ranged from approximately 1 to 10 μm. Grain boundary migration was suppressed by the pinning effect of the fine Al–Fe intermetallic phases dispersed in the α-Al matrix (Figure 3), which led to a relatively fine-grained microstructure even after exposure to 500 °C for 100 h (Figure 8d).

**Figure 9:**
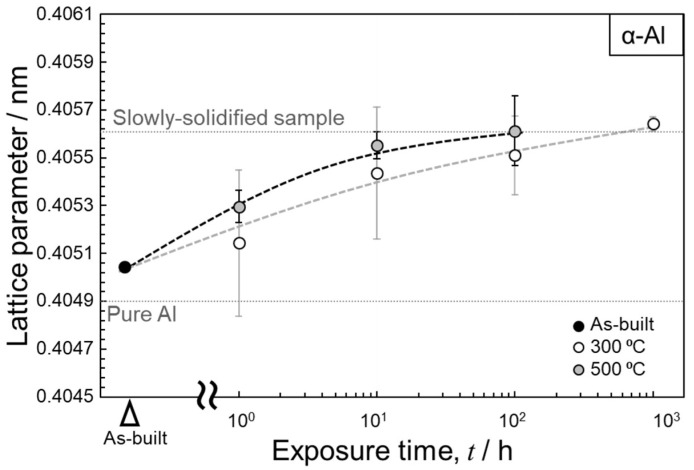
Changes in lattice parameter of the L-PBF-built Al–15%Fe alloy samples as a function of exposure time.

Figure 9 shows the variations in the lattice parameter of the α-Al matrix in the thermally treated samples (300 and 500 °C) as a function of the exposure time. The lattice parameters were calculated using the XRD profiles shown in Figure 2. The scatted values of the measured lattice parameters suggest the inhomogeneous distribution of a solute Fe element in the α-Al matrix due to the complicated microstructures of the L-PBF-processed Al–15%Fe alloy samples. The lattice parameter of the as-built sample was ~0.405 nm, whereas that of the thermally treated specimens increased with the increasing exposure time and almost stabilized at ~0.406 nm. This trend was observed for both sets of the thermally exposed specimens (300 and 500 °C). The atomic radius of Fe (0.127 nm) is smaller than that of Al (0.143 nm) in the fcc structure [33]. Therefore, the observed increase in the lattice parameter could be due to the reduction in the Fe solute in the α-Al matrix upon exposure to elevated temperatures. The saturated value of the lattice parameter (~0.406 nm) is equivalent to that of the fully melted and slowly solidified samples of the used Al–15%Fe alloy powder [21] that solidifies at a low cooling rate of ~0.3 °C/s [34]. These results suggest that the Fe solute content almost achieved an equilibrium state after prolonged thermal exposure. Intriguingly, all the measured lattice parameters of the used Al–15%Fe alloy samples were greater than that of pure Al (0.40493 nm). The large lattice parameters of the α-Al phase in the L-PBF-built Al–15%Fe samples were presumably due to the presence of O solute atoms positioned at interstitial sites in the fcc structure. Composition analyses [20,21] have revealed that the alloy powder contains ~0.25–0.3 mass% O, which indicates that a thin oxide layer is present on the investigated alloy powder particles. These oxide films could dissolve in the alloy melts during L-PBF. STEM characterization [21] has revealed that nanoscale oxide particles can be formed in the microstructure of as-built samples; however, the O content may be partially disbursed in the α-Al phase during solidification, resulting in the expanded lattice in the investigated L-PBF-built samples. A similar trend has been found in Al–2.5%Fe binary alloy samples [35]. However, direct evidence concerning the presence of O solute in the α-Al phase has not been obtained. Therefore, additional characterization experiments must be performed to clarify the state in which O exists in L-PBF-processed Al alloys.

## 4. Conclusions

Variations in the refined Al–Fe intermetallic phases and microstructure of the α-Al matrix in an L-PBF-built Al–15%Fe alloy upon exposure to intermediate temperatures (300 and 500 °C) were examined. The key findings are summarized below.

- The microstructure of the as-built sample had a stable θ-Al_13_Fe_4_ phase localized along the melt-pool boundaries as well as numerous particles of the metastable Al_6_Fe phase surrounded by a nanoscale α-Al/Al_6_Fe cellular structure in the melt pools. The morphology of the θ phase remained almost unchanged even after 1000 h of exposure at 300 °C. The cellular-structured Al_6_Fe phase dissolved in the α-Al matrix, which was followed by the growth of Al_6_Fe-phase particles and the precipitation of the θ phase. The growth of the θ phase was more pronounced at the higher investigated temperature (500 °C).
- Numerous elongated grains with widths of a few tens of micrometers were observed in the α-Al microstructure of the as-built sample, in addition to locally present fine grains. Relatively equiaxed grains were often formed after exposure to 300 °C, particularly in the fine-grained regions. This tendency was more evident at the higher temperature of 500 °C, which led to the formation of a homogenous microstructure. The lattice parameter of the α-Al matrix increased with increasing exposure time, and almost stabilized at ~0.406 nm. The lattice expansion could be associated with the reduction of the Fe solute, which has a smaller atomic radius than that of Al.
